# A Study to Explore the Parental Impact and Challenges of Self-Management in Children and Adolescents Suffering with Lymphedema

**DOI:** 10.1089/lrb.2018.0077

**Published:** 2019-04-22

**Authors:** Christine Moffatt, Aimee Aubeeluck, Elodie Stasi, Roberto Bartoletti, Christine Aussenac, Dario Roccatello, Isabelle Quéré

**Affiliations:** ^1^ School of Social Sciences, Nottingham Trent University, Nottingham, United Kingdom.; ^2^ Department of Vascular medicine, CHU Saint Eloi, University of Montpellier, Montpellier, France.; ^3^ School of Health Science and Medicine, University of Nottingham, Nottingham, United Kingdom.; ^4^ Center of Research of Immunopathology and Rare Diseases—Coordinating Center of the Network for Rare Diseases of Piedmont and Aosta Valley, Turin, Italy.; ^5^ Dermopathic Institute of Immacolata, IDI-IRCCS, Rome, Italy.

**Keywords:** lymphedema, lymphoedema, quality of life, self-management, self-efficacy, parent

## Abstract

***Background:*** Limited research has shown the impact lymphedema has on children and families. The aim of this study was to explore the parental experience of caring for a child or adolescent with lymphedema and the daily challenges of self-management and self-efficacy.

***Methods and Results:*** Participants were recruited during an educational camp for children with lymphedema (*N* = 26). Three individual semistructured focus groups were undertaken in English, French, and Italian with simultaneous translation. Data were analyzed using interpretative phenomenological analysis. Analysis identified four superordinate themes; the journey, treatment management, independence, and psychosocial impact. Ten subthemes were identified: bandaging/compression, professional support, holistic care, fear, self-efficacy, acceptance, friendship, guilt, distress, and hope.

***Conclusions:*** Parental self-management of children with lymphedema is complex and invades many aspects of life. Lack of professional agreement over what constitutes self-management leads to parental confusion and anxiety. Self-management is demanding, and parents are ambivalent to its effectiveness, but choose to persevere through fear of their child's condition deteriorating. Self-efficacy is evident in complex problem solving, despite parents believing that they are not adequately prepared for this.

## Background

### Lymphedema

Lymphedema results from a complex array of developmental defects in the lymphatic circulation that occur *in utero* with some occurring due to genetic abnormalities. More rarely, children may suffer with secondary lymphedema due to lymphatic damage from treatments for cancer or trauma.^1^ Lymphedema may present at any time in childhood, adolescence, and young adulthood. The epidemiology of primary lymphedema affecting children and adolescents is poorly understood with current views indicating that 1 to 1.5 per 10,000 are affected.^2^ Poor professional knowledge indicates that obtaining a correct diagnosis may take many years and is associated with significant parental anguish.^3^ Treatment involves a combination of manual lymphatic massage (a special form of remedial massage), exercise regimes, skin care, compression therapy, and therapeutic education. During the “intensive phase” a large amount of fluid can be removed, and following this treatment, it is replaced by a life-long maintenance treatment that requires patients and families to embrace daily self-management using these techniques.^4^

### Psychosocial impact on families

Research indicates that lymphedema impacts on the lives of children and their families, with greater difficulties occurring during adolescence.^3^ However, little is known of the challenges of providing daily treatment and its impact on family life. The limited research to date has focused on quality of life impact, but has not attempted to compare or contrast the experience of parents from different countries with diverse health care systems and treatment regimes. Recommendations about what constitutes effective self-management in lymphedema have not been empirically established for either adults or children and are the subject of intense international debate.

Research from other diseases indicates that parents and children face great anxiety before diagnosis and live with high levels of uncertainty and isolation throughout the illness.^5–7^

### Disease management and self-efficacy

When faced with a chronic illness such as lymphedema, parents and children must address the issues of engagement with treatment to help control swelling and reduce complications such as cellulitis, while at the same time promoting well-being and normal childhood development. Self-efficacy refers to the subjective judgments made by individuals and families about their capacity and capability to undertake a course of action to achieve a specific goal.^8^ The outcomes people anticipate from treatment depend on many factors, but are significantly influenced by the judgments they make of how well they will be able to perform these activities in different situations and to solve problems.^9^

The aim of this study was to explore the parental experience of caring for a child or adolescent with lymphedema and the daily challenges of self-management. The study was nested within a larger study to explore the enablers and barriers to self-management in children, adolescents, parents, and professionals.

### Research setting

Parents with children and adolescents were recruited while attending an international educational camp for children with lymphedema in Turin, Italy (2017). The sample included parents from Italy, France, Southern Ireland, and South Africa.

### Ethics

Approval for this study was given by the University of Nottingham Faculty of Medicine and Health Science Ethics Committee. Formal ethical approval was not required in Italy as it was not an intervention study. The research was performed in accordance with the 2013 Declaration of Helsinki.^10^ All participants gave informed consent and were made fully aware of their right to withdraw from the study if they wished to do so.

All study information was translated and back translated into the different languages to ensure accuracy with English, French, and Italian parents reviewing all study material.^11^

## Methods

Parents of the children attending the camp were invited to attend a focus group. Group size was determined by the numbers of parents wishing to attend, and therefore, the size was not limited, but ranged from 4 to 12 (Total *N* = 26).

Two researchers moderated the groups, which were simultaneously translated so that parents could participate in their native language (English, French, and Italian). The researchers wore headsets to ensure that they understood the translation. Neither researchers had any relationship to the participants, but held other research roles during the camp.

### Procedure

Following consent, participants were initially introduced to each other and given a chance to become acquainted with the other members of the group. The Focus groups were semistructured with initial questions focusing on the impact of their child's condition on family life. This was followed by discussions about their daily routines of care and their beliefs about their self-efficacy in providing treatment and the attitudes and behavior toward this. Following this, general discussion questions were responsive and tailored to each individual group. Finally, the researchers summarized the main points raised and asked whether participants' thoughts had been summed up adequately and whether anything had been missed.

All focus groups were audio tape recorded and lasted between 1 and 1.5 hours. If participants became distressed during the focus groups, they were offered time out and asked if they wished to discontinue. Emotional support and counseling were made available to all participants if they wished after each event. Both researchers met immediately after each focus group to complete a field diary of initial impressions and reactions that may have been lost if not captured at this point.

### Data analysis

Each focus group session was transcribed verbatim and interpretative phenomenological analysis used.^12^ The first transcript was read several times, the left-hand margin being used to annotate what was interesting and significant about what the respondents said. Once this had been carried out for the whole of the first transcript, attention was returned to the beginning of the transcript with the right-hand margin annotated with emerging themes. These themes were then taken back to the original transcript to validate their existence within the text. Emergent themes were then listed on a sheet of paper and studied for connections between them. All the themes were clustered together to produce a set of superordinate concepts. As the clustering of themes emerged, they were continually checked in the transcript to make sure that they were evident in the primary source material.

The next stage was to produce a coherently ordered table of themes to establish which most strongly captured the respondents' issues or concerns. The clustered themes were given names that represented their overall superordinate theme and an identifier was added in each instance to aid the organization of the analysis and facilitate checking back to the original transcript. During this process, themes were dropped if they did not fit well into the emerging structure or were not very rich in evidence. Themes from the first transcript were then used to ordinate the analysis of subsequent transcripts. As such, repeating patterns were established, but the emergence of new issues was also recognized. Data from each country focus group were then compared across the three transcripts to explore the cultural differences emerging.

To determine reliability, an additional researcher undertook independent thematic analysis of the verbatim transcripts. Both researchers discussed the themes and subthemes they had identified, and agreement was sought when meaning was deemed the same, but the language used was different, so that informed consensus was achieved.

## Results

Participants (*N* = 26) were asked to discuss in as much detail as possible the parental impact of their child's condition and the daily routines and challenges they faced in providing care. Participants' accounts clustered around four superordinate themes; the journey; treatment management; independence; and psychosocial impact.

Tables 1–3 outline the themes and subthemes arising from the data from each country with supporting parent quotes.

**Table 1.** Superordinate Themes (1) and Subthemes (2) with Supporting Quotes (ENGLISH PARENTS) (*N* = 4) (p1–4)

***The Journey (1)*** “I see the fantastic things they are doing in medicine and I appreciate the work that is being done and I will not say hope is dead because I will continue lifting stones and continue my research despite people saying you are wasting your time, I will continue to search while doing what they recommend as well”(p1)	
***Treatment management (1)***	
*Bandaging/compression (2)*	“Well we do bandage and I had one really good physiotherapist for about 6 months, so she is showing me how to do the bandaging and I am doing the bandaging every night even though we don't see any difference”(p3) “I think you want to do is try to help in any way you can and if bandaging is the only thing you can do then you bandage every night, we played games around them, kind of races about rolling the bandage.” (p2) “he doesn't want to wear his stocking today and I'm like tough, I have to wear my bra and I don't want to so you are going to wear your stocking. So you know, I go, there is no-one in this world who is perfect, you've a good brain, you're a good looking guy, you're clever, you know and popular at school, get on with it and I make lists up about what he is good at and I tell him to just go for it. Once his stocking is on, its ok like it's a struggle to get it on”(p1)
*Professional support (2)*	“I have met some fantastic people along the way and it was a Dr who has a company that imports all the bandages so we have become great friends and he often has therapists from all over the world and we often go there and xxx is like the guinea-pig so they try things on him”(p2) “some Dr's are giving me an enormous amount of their time for nothing which is amazing”(p4)
*Holistic care (2)*	“It seems to me that managing lymphedema is not just about bandaging.”(p2) “there is one American physiotherapist who was like you cannot let him go barefoot, you cannot let him go into the sea, we live right across the road from the beach and you know you think, he's not allowed to play outside and I was like no, I can't do that so you know, we go for walks on the beach and he is quite careful if he has a cut” (3)
*Fear (2)*	“I'm afraid it could go into the arms and then it would be both legs, arms and I was told not to do football by the hospital but I am going, he has to live, he wants football and I said tell me the things he can do and they said trampoline and swimming and I said fine but he wants football” (p1)
***Independence (1)***	
*Self-efficacy (2)*	“He does it by himself now because he is x and he is almost out of the house and he manages by himself” (p2)
*Acceptance (2)*	“it is just something we have to accept” (p1)
***Psychosocial impact (1)***	
*Friendships (2)*	“he has lots of friends is really popular… but he gets teased and I can see that hurts him” (p1) “he was really bullied one time at school he didn't want to go at all we had to go and really tell the school to solve it now he is better…but he still likes to be alone” (p2)
*Guilt (2)*	“you feel guilty and because of this you do what the Dr's say, no matter what and actually the guilt wore off as well and I felt it wasn't something I had done and even if it was something I had done” (p3)
*Distress (2)*	“its lonely, there are no other parents really to talk to (crying)” (p1)
*Hope (2)*	“I've been told there's no cure and I accept that but I hope to think there will be” (p1)

**Table 2.** Superordinate Themes (1) and Subthemes (2) with Supporting Quotes (FRENCH PARENTS) (*N* = 10) (p1–10)

***The Journey (1)***	
“It is very hard, sometimes if feels like an impossible challenge, it is a long journey, we have to see people in X, see the therapist and it is a journey and it is an ordeal and it feels this way, every day without exception. Even when you accept it, it is a heavy weight, there are different ways of managing it and living with the stress” (p1)	
**Treatment management (1)**	
*Bandaging/compression (2)*	“You have to wear your bandages every day and you have to roll up the bandages, it's a core task that needs doing every day and it is time consuming” (p2)
*Professional support (2)*	“To find the right specialist who will take care of my child. We have to establish a good link, a good bond.”(p1) “You need to have the right doctor that you can trust to help you” (p5)
*Holistic care (2)*	“these obstacles affect our whole lives and it is not for me to say but for the doctor it can be about volume but that is not all it is, it is not just volume reduction” (p3)
*Fear (2)*	“we are afraid, we are afraid of infection and that happened last night and we had to go to the doctor and he is now on antibiotics from last night—we cannot solve this problem ourselves.” (p2)
***Independence (1)***	
*Self-efficacy (2)*	“It was very good for her and when the opportunity came for her to go on this trip, this is when she learnt how to use her stockings and to be independent and comfortable” (p7) “She doesn't always want me to help her …. She wants to do everything on her own so” (p8)
*Acceptance (2)*	“What is really important in this is accepting the condition. To feel like anyone else” (p3)
***Psychosocial impact (1)***	
*Friendships (2)*	“I worry that she doesn't want to mingle with it, that is makes her different and is difficult to make friends” (p4) “My daughter afraid to go to high school because of the other students, what they might say to her or look at her, mocking her, it is very difficult.”(p6) “I have found that other teenagers attempt to protect my child form problems in the class and if somebody tried to tease them, they protect them” (p9)
*Guilt (2)*	“when I found out it could not be cured that was the worst of it and I thought about if I had done anything when I was pregnant, maybe drink wine and that made me feel guilty.”(p2) “My son when he was 8 it was difficult to watch him struggle and you feel guilty like there is more you should do, that you wish it was you and not them.” (p1)
*Distress (2)*	“For me it was very difficult, when I realized it was a lifelong problem, I cried. It is good to have the support of friends and the X team. It can also be really hard for the wider family to accept but we have been helped quite a lot by the team…. And that is all. (crying).” (p7)
*Hope (2)*	“My child is 17 years old and he had a girlfriend now and that makes us feel better. That he is managing to get on with life, that he is doing things that are normal, that he will have a life, that the worst is gone.” (p10)

**Table 3.** Superordinate Themes (1) and Subthemes (2) with Supporting Quotes (ITALIAN) (*N* = 12) (p1–12)

***The Journey (1)***	
“at the beginning it was very hard because there was no doctor. There are no hospitals that can give you information about the disease or tell you what to do” (p2) “We have been to different doctors and everyone was saying something different. But we need answers. We want to understand. They were just saying that the leg was functioning and not sore—if it works then there is no problem” (p7)	
***Treatment management (1)***	
*Bandaging/compression (2)*	“Being able to do the bandaging and massaging. Life became quite normal” (p3) “We have not started yet with the stocking. When we used, it did not give enough visible result.”(p7) “Once you know then your life becomes easier. However, every single day you have to live with the disease. When you wake up, the first thing you need to do, is to put on the garment. You need to do this every single day.”(p10)
*Professional support (2)*	“Though I knew I had LE, (the name and what it was) I was scared when I had the disease and it took me 3 years to find the people and find out what to do and how. Then I met the people and I learned how to do it and I taught it to my mum. And we do the daily routine and we reached the result of having equal sized legs. We reached excellent results. But you really need to have the right people to reach this goal.”(p12)
*Holistic care (2)*	“We have many problems the doctors do not understand this they just focus on the swelling we have to find our own solutions” (p1)
*Fear (2)*	“Until we face the point of no return then we can see the swelling is increasing. When you see the problem is stable you don't care/worry. Then when you see that the swelling is increasing you start to care/worry.” (p2) “Following what X is saying the psychological effect is heavy. Just the fear of something will happen stops you. If you accept you have this disease, then you can live a normal life as the progression is not fast. Without living in constant fear. The worst fear is the progression.” (p8)
***Independence (1)***	
*Self-efficacy (2)*	“Seeing that it is working it gives you motivation” (p4) “We manage not to do all these treatments perhaps because the problem is not as big/evident. For her it is not a problem as she has always seen herself like this. X does not bother too much about managing the problem. It is just us as adults we see the problem.” (p6) “It is not easy as they are teenagers. She is very stubborn. We tell her to do this and that. The doctor told us to proceed slowly trying to convince her not to force her. Small steps. We try to make her understand. It is not easy. We cannot have a precise plan. Confused times.”(p5)
*Acceptance (2)*	“And since the 4–5 years since he was born it regressed. And now it is barely manifested. He is very lucky.”(p3)
***Psychosocial impact (1)***	
*Friendships (2)*	“Before knowing people in this group they did not know anyone. But in this camp we can support each other. Between us we share/exchange experience and feel better. And we exchange the information we find in different areas of Italy and we cope with life knowing we are not alone.” (p6) “Information—there is very little. Italian proverb: „if everyone add a piece then we have a loaf”(p10)
*Guilt (2)*	“And for me this was a shock. Because back in the days I was told I was just unlucky. It was not even conceived that it could be hereditary I feel such guilt this is my fault and I never knew.” (p9)
*Distress (2)*	“Someone told to X to cover yourself/cover your disease because you scare me.” And she answered, “then don't look at me.”(p12)
*Hope (2)*	“We share hope together as parents and the camp helps us we are so happy so grateful for this.” (p11)

### The journey

The English-speaking parents lived in South Africa and Ireland and reported a tortuous journey through a maze of different health professionals. Obtaining a correct diagnosis took many years and involved being given an incorrect diagnosis and advice on treatment on many occasions. This in turn caused parental uncertainty and anxiety and led them to a continuous and exhausting search for the correct information, which they felt ill-equipped to interpret. Poor professional knowledge resulted in some parents being told that their child would naturally recover over time only to find that this did not occur leading to a further eroding of trust. One mother did not accept that the current level of treatment was all that could be offered and was seeking advice from many experts in her search for a “cure.” The journey involved constant negotiation and advocacy in many aspects of life, including health care and schooling. Parents reported the loneliness of the journey with no access to other parents, but that they had no choice but to continue the journey for the sake of their child.

The French parents present a different picture. All the children at the camp are managed by a specialist service in France. Access to a high level of expertise and a sense of belonging are of critical importance to the parents. Unlike the English parents, they do not refer to a search for information or uncertainty about their child's diagnosis. Despite this, the journey to be referred into the service was often difficult and protracted. The French parents showed strong emotional reactions and expressed fear and uncertainty of the future, despite access to specialist care. The parents report the importance of the wider team, including therapists and psychologists, in addressing complex issues. While highly valuing the team, the distance to attend the service was a significant burden for some. Parents value their ability to share and support each other through the support group and the relationships that have been established.

The evident lack of specialist services and poor professional knowledge in Italy is similar to that encountered by the English parents. They face a long and complex struggle to find information or gain a diagnosis causing parental distress and uncertainty. The strong and beneficial bond they had between the parents and the professionals caring for them was very evident.

### Treatment management

English parental attitudes to the current recommendations of treatment vary. Some expressed frustration while others felt they had reached an equilibrium. All parents reported that they continued treatment because there was no other option available despite not seeing an effect. For many, the advice given was dogmatic and required strict daily regimes. Parents varied in their adaptability of treatment and their willingness to allow their child's participation in sports and other activities. Fear was a major factor influencing these choices and parents experimented with the degree of freedom they allowed. Positive experiences reinforced their relinquishing of control. Treatment was considered burdensome for all parents, impacting on family life and causing a financial burden.

French parents' experience demonstrated that treatment is a burden to family life. The parents report some flexibility in their approach to daily treatment and they stress the importance of allowing the child to learn the techniques and to relinquish control. The French parents not only speak of the value of access to advice from the service when problems occur but also express fear about complications such as cellulitis. French parents refer to volume reduction as the important outcome for doctors, which differs from their views, which focus on leading a normal life and a strong emphasis on promoting independence as the primary goal.

The Italian experience of treatment focuses on the development of skills and the control of the swelling as a key goal. Achieving control of the swelling led parents to consider their life as normal, despite the daily challenges and resistance of their children at times to cooperate with treatment.

### Bandaging/compression hosiery

Parents from all countries recognized the value of skilled professionals who were able to teach them the correct use of compression and how to adapt this for their child. Bandaging at night was a key priority for many. Children were often frustrated at this and parents sought to normalize this with routines and fun activities. Parents continued with compression despite seeing no benefit. Access to compression in countries such as South Africa and Ireland was often difficult with parents having to seek ways to obtain materials that were not readily available. Daily application of compression hosiery was less difficult, but often resisted by the child. Continuation of compression was driven by the fear that stopping would lead to deterioration or extension of the lymphedema into other areas of the limb or body.

### Professional support

Parents from all countries recognized skilled practitioners who were both knowledgeable and willing to problem solve on behalf of their child. Parents were clear that treatment was much greater than the use of compression and that it involved a tension between allowing the child a normal life and the burden of treatment. French parents stress the importance of their child's independence, while the English parents focus on the dilemmas of understanding the risk of normal activities, which allow greater freedom. A key issue for them is whether they perceive the lymphedema to worsen because of their parental decisions. Distinct differences emerged between those able to access a multidisciplinary team and those seeking treatment or managing in isolation. Italian parents balance the evident risk of delaying treatment with a desire for their child to undertake normal activities and to prevent emotional distress.

### Holistic care

The parental distress manifested during all focus groups indicated the need for a holistic approach to care that addressed the psychological and social issues they faced. English parents reported that this aspect was absent from most of their interactions with professionals, while the French parents reported that they were given an ability to offload stress to the team as well as to each other. The Italian parents also strongly valued the support they had for each other and the value of the relationships with the therapists treating their children.

### Fear

Fear was discussed in all focus groups and varied from extreme in a parent seeking for a cure to very low in those whose children's conditions was stable. Fear was linked to concerns over the deterioration of the condition and how this may impact on their transition into adulthood. Lack of access to skilled professionals who understood the condition increased the level of fear. Parental uncertainty over the future was evident and the realization that professionals did not have all the answers. They expressed the value of meeting and sharing their fears together during the camp, thereby breaking their sense of isolation.

### Independence

#### Self-efficacy

Parents did not see themselves as self-efficacious, despite the evidence that they were often able to solve complex problems and adapt treatment. They saw the importance of their child taking on treatment and becoming independent and able to use effective problem solving. There was evidence for some that success with treatment was a motivating factor. The search for cure or complete control also motivated some parents to search for doctors who they believed could eradicate the lymphedema despite being told evidence to the contrary. Novel treatments such as diets that were not recommended by professionals were also discussed by some.

#### Acceptance

Parental acceptance of their child's situation was complex and influenced by where they were on the illness journey and the array of other medical and psychosocial issues they faced. Their judgment about the level of stability of the swelling influenced their acceptance of the situation, but was often found after many years of struggle and adjustment.

#### Friendships

Parents expressed a deep concern for their child to experience friendships and normal adult relationships. A number of children shared their experience of addressing bullying at school and the way they addressed this. Building confidence was important as was encouraging their child to consider the positive aspects of life.

#### Guilt

Parents from all countries expressed guilt over their child's condition. This was particularly painful if they had a genetic form, but was also evident in those without. Mothers expressed concern that they may have unwittingly caused this during pregnancy. Fathers talked of the guilt they felt of their children being unable to fully participate in childhood activities. Parental guilt was pronounced in those whose children were bullied at school and in children who struggled to manage with their lymphedema.

## Discussion

The aim of this study was to explore the parental experience of caring for a child with lymphedema and the daily challenges of self-management. Analysis of the data produced four superordinate themes; the journey, treatment management, independence, and psychosocial impact. An additional 10 subthemes were identified: bandaging/compression, professional support, holistic care, fear, self-efficacy, acceptance, friendship, guilt, distress, and hope.

The difficulties of the journey to diagnosis and treatment were very evident in this study and have been reported in children with lymphedema in the United Kingdom a decade ago.^3^ Research with children with cystic fibrosis showed that a misdiagnosis led to increased parental anxiety, guilt, anger, and mistrust of the medical profession.^13^

This study indicated that the lack of dedicated lymphedema services and poor evidence base for treatment are major factors that negatively influence the parent experience. Lack of reliable information led parents whose children were not treated in a dedicated multiprofessional service on a constant quest for answers. Parents faced an inability to understand the complex and rapidly evolving information that they were able to identify and to understand this in the context of their child's condition.

Uncertainty was a major factor for all parents, but was more acute in those searching for expert care. Uncertainty about the future of their child was pervasive and caused evident emotional distress that was manifested during the focus groups. The importance of uncertainty in managing chronic illness has been known for many decades, Koocher and O'Malley^6^ underscored the importance of uncertainty when they coined the phrase, “The Damocles Syndrome,” to characterize the pervasive fears of illness recurrence, death, and long-term consequences of treatment, which accompany the promise of life-saving treatment for survivors of childhood cancer. Koocher^14^ describes uncertainty as “probably the greatest single psychological stressor facing the patient with a life-threatening illness.” Stewart and Mishel^15^ reported parental psychological distress to be the single most commonly reported consequence of parental uncertainty, and it has been shown to dominate parents' experiences with ill and disabled children in acute, critical, outpatient, and home-care settings.^16–20^ Although the intensity of uncertainty may diminish during periods of relative stability, research has shown that a subset of parents are at an increased risk of developing depression and anxiety shortly after their child's diagnosis, with a select subset continuing to exhibit distress symptoms even after treatment is completed.^21,22^

Uncertainty is heightened when the condition is an “orphan” illness not belonging to one medical specialty or where access to specialist care is absent, a common problem for lymphedema. Illness uncertainty has also consistently emerged as a salient predictor of adjustment outcomes,^15^ including parents of children with Type 1 diabetes,^23^ cancer,^24^ and asthma.^25^

### Self-efficacy

Parents from all countries when asked whether they felt self-efficacious did not see themselves in this way. However, the data show a more complex issue. There was evidence of problem solving and a belief that they could influence their child's illness, improve their overall wellbeing and find solutions for the daily treatment challenges they faced. Success was celebrated and was a strong motivator to persevere. Parents of adolescents strove to transfer self-management to their children. They acknowledged that this was difficult and often was undertaken inconsistently, especially when they felt their child had not come to accept their condition and when the degree of swelling influenced the child's daily life. Parents were aware that a “normal limb” was of great importance for aesthetic reasons as well as the challenges of obtaining fashionable shoes and clothes. Parents focused on the need for their child to undertake a normal life and participate in activities. This was set against the fear of complications if they undertook activities they appraised as high risk. The situation was exacerbated for parents who had been rigidly taught by professionals.

Achieving stability of the swelling and lack of complications allowed parents to be more relaxed in their attitudes toward treatment. Deterioration of swelling was occurring in a few children during the camp. This led to high levels of parental anxiety and fear. These parents not only sought the advice of the clinicians present but also shared the issues with other parents. They expressed the importance of the shared emotional and tangible support and solutions that other parents could provide, many of whom had gone through a similar experience. They talked of the importance of the multiple levels of knowledge and support available through the camp and breaking the cycle of isolation.

Research has shown that positive outcomes act as incentives and drive adherence to treatment, while negative outcomes act as disincentives. The belief people have about their self-efficacy also influence whether they think erratically or strategically and optimistically or pessimistically.^26^ Self-efficacy has been shown to be an important predictor of the following: how much effort and commitment they will give to a task; the degree of tenacity in the face of obstacles; the quality of their emotional life; the degree of stress and depression they experience in taxing environments; lifestyle choices; and levels of adherence to treatment. Levels of self-efficacy may influence parental behaviors toward adolescents with chronic illness. Research has shown that those with high levels of self-efficacy show greater adherence to recommendations for diabetes management. Improved self-efficacy in children with varying degrees of congenital cardiac malformations have been shown to improve physical activity, which in turn mediates the attitudes of both parents and cardiologists about treatment and the degree of risk they can safely undertake.^27^

The expression of strong emotions was evident in all focus groups with many mothers and fathers crying during the discussion. Despite this, the parents did not wish the sessions to stop describing them as a cathartic experience that they had rarely been offered. It appeared to draw the parents together in support of each other.

Parental distress managing a child with a chronic illness may lead to psychological problems such as depression and anxiety. Research in children with learning disability found that a significantly high proportion of mothers (89%) had anxiety, depression, or both anxiety and depression together compared with fathers (77%) (*p* < 0.05). Among mothers, 35% met the criteria for anxiety, 40% for depression, and 13% for both anxiety and depression. Among fathers, 42% had anxiety, 31% depression, and 3% both anxiety and depression.^28^ Post traumatic stress disorder (PTSD) was seen in a systematic review of parents of children with chronic illness. The pooled PTSD prevalence was 19.6% in mothers, 11.6% in fathers, and 22.8% in parents in general (*p* < 0.001). The prevalence in the healthy control group of mothers was only 4.2% (*p* < 0.001).^29^

### Study limitations

This study has several limitations. It only represents the views of the parents who agreed to take part in the focus groups during an international educational camp. As such, they may not reflect the views of other parents who do not access such camps and who may be uncomfortable sharing within a focus group setting. In addition, the parents are drawn from countries with different health care settings, and the context of care and culture is likely to differ affecting the overall generalizability of the research. However, despite this, the study shows many of the issues raised were common across the groups, indicating that this is an important issue that must be addressed.^30,31^

## Conclusion

The purpose of investigating the experience of parents managing a child with lymphedema is to give greater understanding of the challenges they face and their own sense of self efficacy. The focus groups provided a forum that parents could talk in-depth about the issues that enhanced and compromised their life quality and impacted on the self-management of their child. Moreover, although the focus group conversations appeared to flow freely, it is not known whether there were issues that parents might not have wanted to discuss in an open discussion in front of their clinical teams. These issues might have been disclosed in an individual interview setting. Further work needs to be carried out to more fully investigate the self-management of children and adolescents with lymphedema and to design and evaluate interventions that may be useful in supporting and evaluating their needs.

## Implications for Clinical Practice

The research has shown an urgent need to address the following issues:

- Develop multidisciplinary services for children in all countries
- Provide clarity on self-management strategies in lymphedema
- Develop accessible and reliable sources of information for parents
- Educate health professionals about the reality of managing a child or adolescent with lymphedema
- Develop and evaluate low intrusion self-management programs
- Develop new compression that is easier to use and aesthetically acceptable to children and adolescents
- Promote normal childhood growth and adaption
- Develop patient support systems for parents and children
